# COVID-19 vaccine-induced lymphadenopathies: incidence, course and imaging features from an ultrasound prospective study

**DOI:** 10.1007/s40477-022-00674-3

**Published:** 2022-05-04

**Authors:** Valeria Romeo, Arnaldo Stanzione, Divina D’Auria, Ludovica Fulgione, Fabio Giusto, Simone Maurea, Arturo Brunetti

**Affiliations:** Department of Advanced Biomedical Sciences, University of Naples “Federico II”, Via S. Pansini, 5, 80131 Naples, Italy

**Keywords:** COVID-19 vaccine, SARS-Cov-2, Axillary lymphadenopathy, Ultrasound, Shear-wave elastography

## Abstract

**Aims:**

lymphadenopathy can occur after COVID-19 vaccination and when encountered at ultrasound examinations performed for other reasons might pose a diagnostic challenge. Purpose of the study was to evaluate the incidence, course and ultrasound imaging features of vaccine-induced lymphadenopathy.

**Methods:**

89 healthy volunteers (median age 30, 76 females) were prospectively enrolled. Vaccine-related clinical side effects (e.g., fever, fatigue, palpable or painful lymphadenopathy) were recorded. Participants underwent bilateral axillary, supraclavicular and cervical lymph node stations ultrasound 1–4 weeks after the second dose and then again after 4–12 weeks in those who showed lymphadenopathy at the first ultrasound. B-mode, color-Doppler assessment, and shear-wave elastography (SWE) evaluation were performed. The correlation between lymphadenopathy and vaccine-related side effects was assessed using the Fisher’s exact test.

**Results:**

Post-vaccine lymphadenopathy were found in 69/89 (78%) participants (37 single and 32 multiple lymphadenopathy). Among them, 60 presented vaccine-related side effects, but no statistically significant difference was observed between post-vaccine side effect and lymphadenopathy. Ultrasound features of vaccine-related lymphadenopathy consisted of absence of fatty hilum, round shape and diffuse or asymmetric cortical thickness (median cortical thickness of 5 mm). Vascular signal was mainly found to be increased, localized in both central and peripheral regions. SWE showed a soft cortical consistence in all cases (median value 11 Kpa). At follow-up, lymph-node morphology was completely restored in most cases (54/69, 78%) and in no case lymphadenopathy had worsened.

**Conclusion:**

A high incidence of vaccine-induced lymphadenopathy was found in a population of healthy subjects, with nearly complete regression within 4–12 weeks.

**Supplementary Information:**

The online version contains supplementary material available at 10.1007/s40477-022-00674-3.

## Introduction

With increasing COVID-19 vaccination rates in the general population, lymphadenopathy have been frequently observed as an incidental finding in different imaging modalities [1–3]. In particular, the mRNA COVID-19 vaccine by Pfizer-BioNTech (Comirnaty) has been associated to post-injection regional lymphadenopathy [4–6]. Indeed, axillary nodal reactivity has been long observed on ultrasound (US) as a side effect of vaccinations, particularly for those that evoke a strong immune response, although their reported incidence was relatively low [7–9]. Over time, the occurrence of vaccine-related axillary, and supraclavicular lymphadenopathy presenting both as a palpable mass or as an incidental finding during routine breast imaging after COVID-19 vaccination, has quickly grown, which allowed to detail their clinical as well as sonographic features. To date, enlarged axillary nodes can be frequently found at US examination performed after both first and second dose of Comirnaty, despite the phase III trial of the same vaccine reported their occurrence for only 0.3% of recipients [10]. Furthermore, according to the Society of Breast Imaging recommendations, for those patients, lymphadenopathy have been only considered “unsolicited adverse event” with 58 more cases in the vaccine group than the placebo group (64 vs 6 respectively) [11].

However, recent data suggest that enlarged lymph nodes (LN) after COVID-19 vaccination should be considered reactive in the first instance, occurring due to the immune system overstimulation [12]. There has been an increase in the number of papers showing their incidence and prevalence, such as the recent results reported with the analyses of over 160 cases in a Specialized Breast Imaging Clinic in Israel [12]. Nevertheless, to the best of our knowledge, content of published radiological reports are still lacking in temporal monitoring, so only limited evidence is currently available. Considering the magnitude of the COVID-19 vaccination campaign, and thus the expected occurrence of vaccine-related lymphadenopathy on a large scale, it is mandatory to not only assess its incidence but particularly determine its course, which would provide important information for patient management, especially in the oncologic setting. US is the imaging modality of choice for the assessment of axillary lymphadenopathy, through the assessment of lymph-node anatomy (B-mode), vascularization (color-Doppler) and stiffness (elastography); this last tool has been recently boosted by the introduction of shear-wave elastography (SWE), a new technique which provides quantitative stiffness data without using transducer pressure. So far, SWE proved to be promising for the differentiation of benign and malignant LN in different anatomical sites, including axillary and head and neck [13, 14]. Therefore, aim of this study was to prospectively evaluate the incidence, course and imaging features of Comirnaty-induced lymphadenopathy using B-mode US, color-doppler and SWE.

## Materials and methods

### Patient population

Between February and July 2021, during the Italian vaccination campaign, healthcare professionals who were scheduled to receive Comirnaty vaccine were invited to join this prospective study, undergoing a US examination of the axillary, subclavicular and cervical regions. Inclusion criteria were: > 18-year-old healthy subjects, with no previous pathological conditions, as assessed by medical history and physical examination, with no prior COVID-19 infection.

Subjects who were missed at follow-up or with subsequent diagnosis of COVID-19 during the study period were excluded. The study population initially included 95 healthcare professionals of our Institution who received Comirnaty during the study period (between January 2021 and March 2021). Of these, six subjects were excluded due to the following reasons: diagnosed with COVID-19 (*n* = 1); did not complete the vaccination schedule (*n* = 2); missed during follow-up (*n* = 3). Thus, a total of 89 subjects were finally included, of whom 76 (85%) females and 13 (15%) males with a median age of 30 years (Interquartile range -IQR- 6). A flow-chart illustrating the patient selection process can be found in Supplementary Materials.

### Clinical data collection

Clinical information including age, sex, family history, vaccination dates and as well as the side of the vaccine administration were collected. Then, all observed vaccine-related side effects have been recorded, including local pain at the injection site, fever, fatigue, headache, muscle or joint pain and, finally, occurrence of palpable or painful lymphadenopathy.

### US examinations

US examinations were performed using a GE Logiq S8 scanner equipped with a high-frequency linear probe (11–15 MHz). Participants underwent bilateral axillary, supraclavicular and cervical LN stations exploration shortly after the second dose 1–4 weeks (Time point 1). Follow-up US examinations were performed after 4–12 weeks (Time point 2) in patients who showed isolated or multiple lymphadenopathy at the first time point. B-mode, color-Doppler assessment, and shear-wave elastography evaluation were performed.

LN were first analyzed using B-mode US to assess LN shape (round, oval, irregular), size in terms of long/short axis, the presence/absence of fatty hilum and to measure the cortical thickness, considered as abnormal if greater than 3 mm [15]. LN cortex was also morphologically evaluated to identify the presence of focal/diffuse lobulations.

Thereafter, color-Doppler US was performed to analyze LN vascularization, in terms of central vascularization, with single hilar vascular signal; peripheral vascularization, characterized by vascular signal detected at LN periphery; and irregular/anarchic vascularization. SWE was then used using a dedicated linear US probe (9 MHz), positioned at the level of the largest/highest suspicious LN. No compression was applied for the generation of colored maps reflecting tumor stiffness, with color-scale intended as hard (red) and soft (blue). Circle ROIs were then placed within LN cortex to quantify its stiffness, express as Kilopascal (KPa). Two ROIs were placed to measure the stiffness of thickened cortex (E1 and E2) at the level of the hardest areas, as detected through the colored map.

### Statistical analysis

The Shapiro–Wilk test was used to assess the distribution of continuous variables, which are presented as mean and standard deviation or median and interquartile range (IQR), as appropriate. On the other hand, categorical variables are presented as count with percentages.

The incidence of lymphadenopathy after COVID-19 vaccine was calculated. The correlation between the occurrence of lymphadenopathy and clinical symptoms after receiving the second vaccine dose was assessed using the Fisher’s exact test. A *p* value ≤ 0.05 was considered statistically significant. Statistical analyses were performed using MedCalc for Windows, version 20.014 (MedCalc Software, Ostend, Belgium).

## Results

Axillary or cervical post-vaccine lymphadenopathy ipsilateral to COVID-19 vaccination injection site were found in 69/89 participants (79.3%). Of these, 37 (53%) developed a single axillary lymphadenopathy, whereas 32 (46%) also developed multiple lymphadenopathy at cervical (*n* = 4) and subclavicular (*n* = 3) stations. Sixty (87%) of 69 subjects presented with clinical symptoms, such as fever (33 of 69, 47%), headache (14 of 69, 20%), ipsilateral armpit pain (31 of 69, 45%), palpable axillary lump (9 of 69, 13%), fatigue (22 of 69, 32%). However, pathological findings can also be found in asymptomatic subjects, as occurred in the remaining nine cases. Indeed, while a trend can be appreciated, no statistically significant difference was observed between the proportion of subjects with any given symptom and the proportion of subjects with post-vaccine lymphadenopathy. Table 1 shows the prevalence of different post-vaccine symptoms in relation to the occurrence of lymphadenopathy.

**Table 1:** Distribution of symptoms between subjects with and without post-vaccine lymphadenopathy

	Without lymphadenopathy (*n* = 20)	With lymphadenopathy (*n* = 69)	*p*
Fever	7	33	0.44
Headache	2	12	0.73
Muscle pain	7	16	0.38
Armpit pain^a^	0	11	0.06
Palpable axillary lump^a^	0	9	0.20
Fatigue	7	19	0.58
No symptoms	8	17	0.26
One symptom	5	22	0.13
Two or more symptoms	6	31	0.31

^a^Ipsilateral

**Fig. 1:**
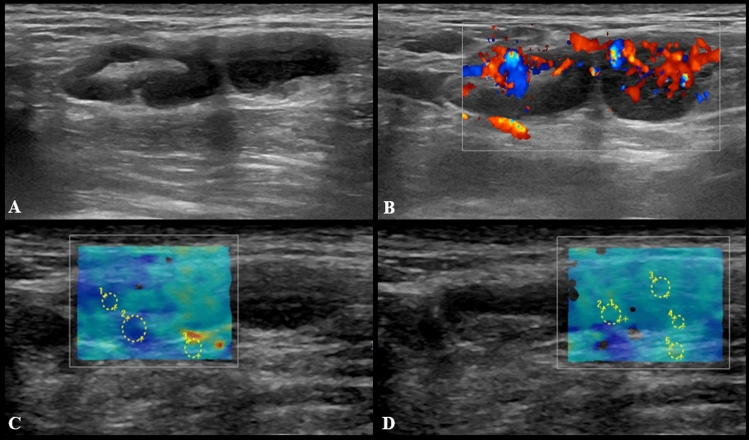
US examination performed 3 days after receiving the second dose of vaccine. Multiple enlarged (maximum diameter 18 × 7 and 14 × 8 mm) axillary lymph nodes with preserved fatty hilum and diffuse cortical thickening (4.5 and 5 mm) are shown in **A**, with vivid hilar and cortical vascularization (**B**) and soft consistence at shear wave that can be appreciated in **C** (*k*1 = 8 kPa, *k*2 = 6 kPa, *k*3 = 25 kPa) and **D** (*k*1 = 19 kPa, *k*2 = 17 kPa, *k*3 = 27 kPa)

**Fig. 2:**
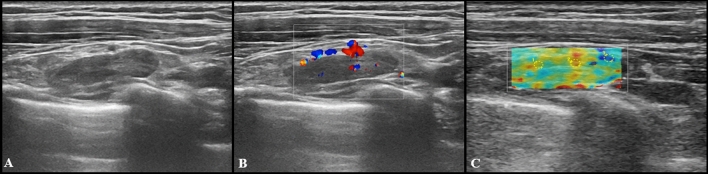
US images from a 27-year-old woman 2 days after receiving the second dose of vaccine: a subclavicular lymphadenopathy (24 × 6 mm) with hilar vascularization was found (**A** and **B**). Shear wave elastography showed soft-intermediate consistence (**C**: *k*1 = 17 kPa, *k*2 = 35 kPa, *k*3 = 13 kPa)

US imaging features of pathological LNs consisted of hypoechoic round or oval nodes as well as LN with preserved fatty hilum but increased (> 3 mm) diffuse or asymmetric cortical thickness (Fig. 1). The median cortical thickness of abnormal LNs after second dose vaccination was 5 mm (IQR = 3) and in 12 (17.3%) participants enlarged LNs had no visible fatty hilum. Short and long axis of abnormal LNs were 7 mm (IQR = 3) and 15 mm (IQR = 9), respectively. Vascular signal was mainly found to be increased, localized in both central and peripheral regions (Fig. 2). SWE showed a soft cortical consistence in all cases, with quantitative E1 ranging between 3 and 40 Kpa (median value 11 Kpa with an IQR of 8,5) and E2 between 4 and 56 kPa (median value 11 kPa with an IQR of 5).

**Fig. 3:**
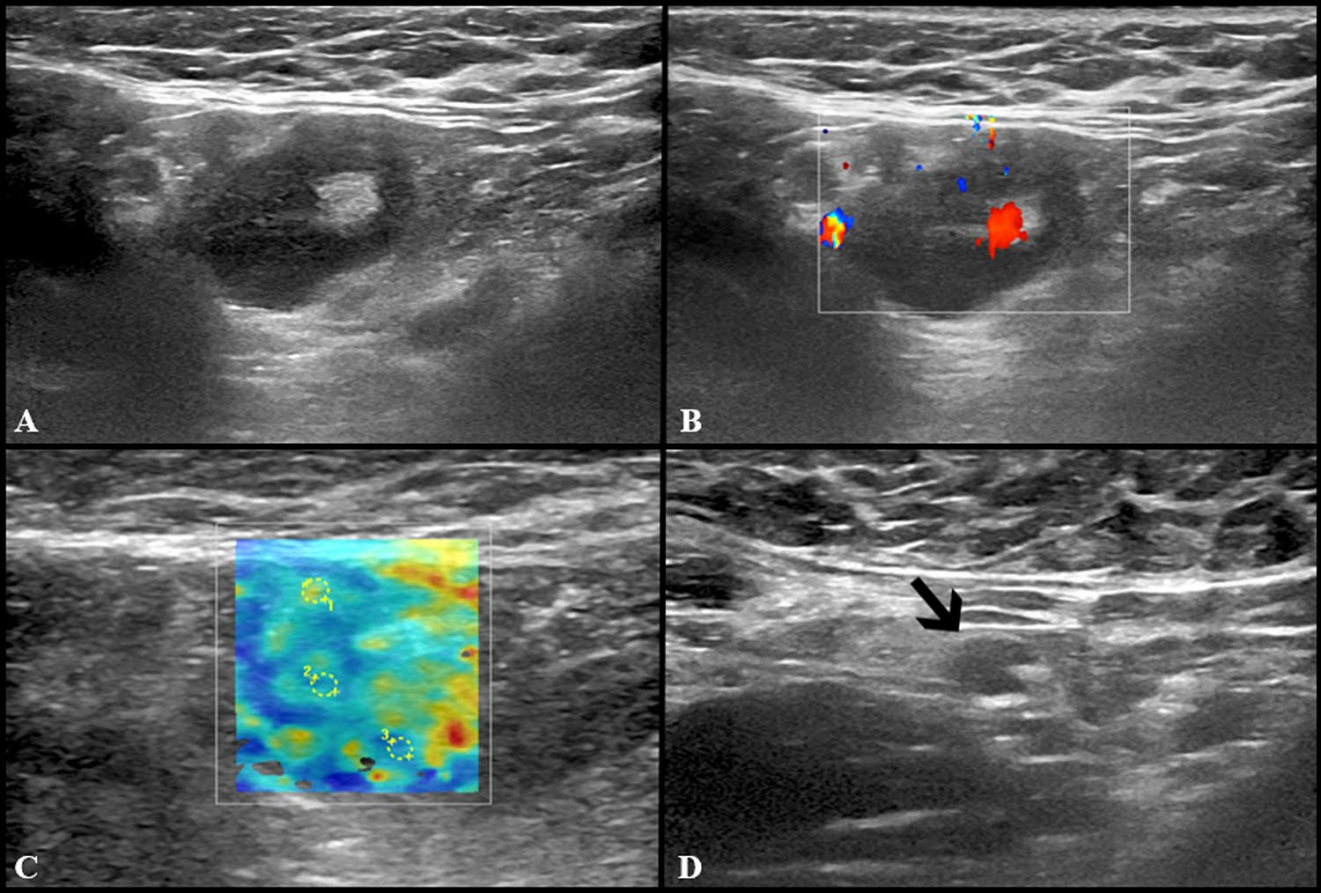
Additional images from the same subject reported in Fig. 2: an enlarged axillary lymph node (17 × 11 mm) with fatty hilum but diffuse cortical thickening (10 mm) and hilar vascularization (**A** and **B**) was detected. Shear wave elastography showed soft-intermediate consistence (**C**: *k*1 = 35 kPa, *k*2 = 17 kPa and *k*3 = 13 kPa). At the follow-up US scan (**D**) performed after 4 weeks lymph node size reduction and normalization of cortical thickness were observed (black arrow)

**Fig. 4:**
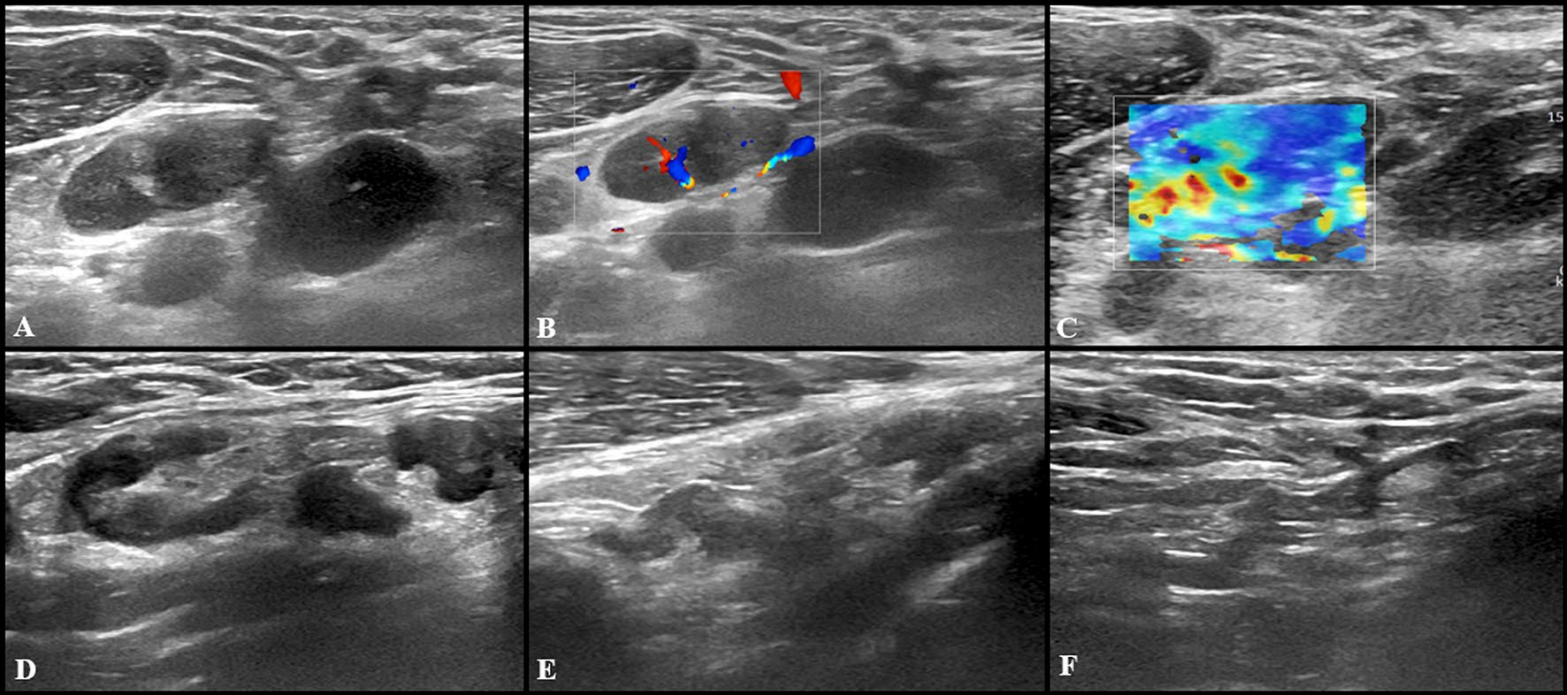
US examination performed in a 27-year-old woman 3 days after receiving the second dose of vaccine: axillary lymph nodes (maximum diameter 14 × 9 mm) with fatty hilum but diffuse cortical thickening (maximum 10 mm) (**A**) and vivid hilar and cortical vascularization (**B**) were detected. Shear wave elastography (**C**) US examination showed soft-intermediate consistence. Follow-up images collected after 4 (**D**), 8 (**E**) and 12 (**F**) weeks show the progressive reduction in size of lymph nodes as well as cortical thickness normalization

**Fig. 5:**
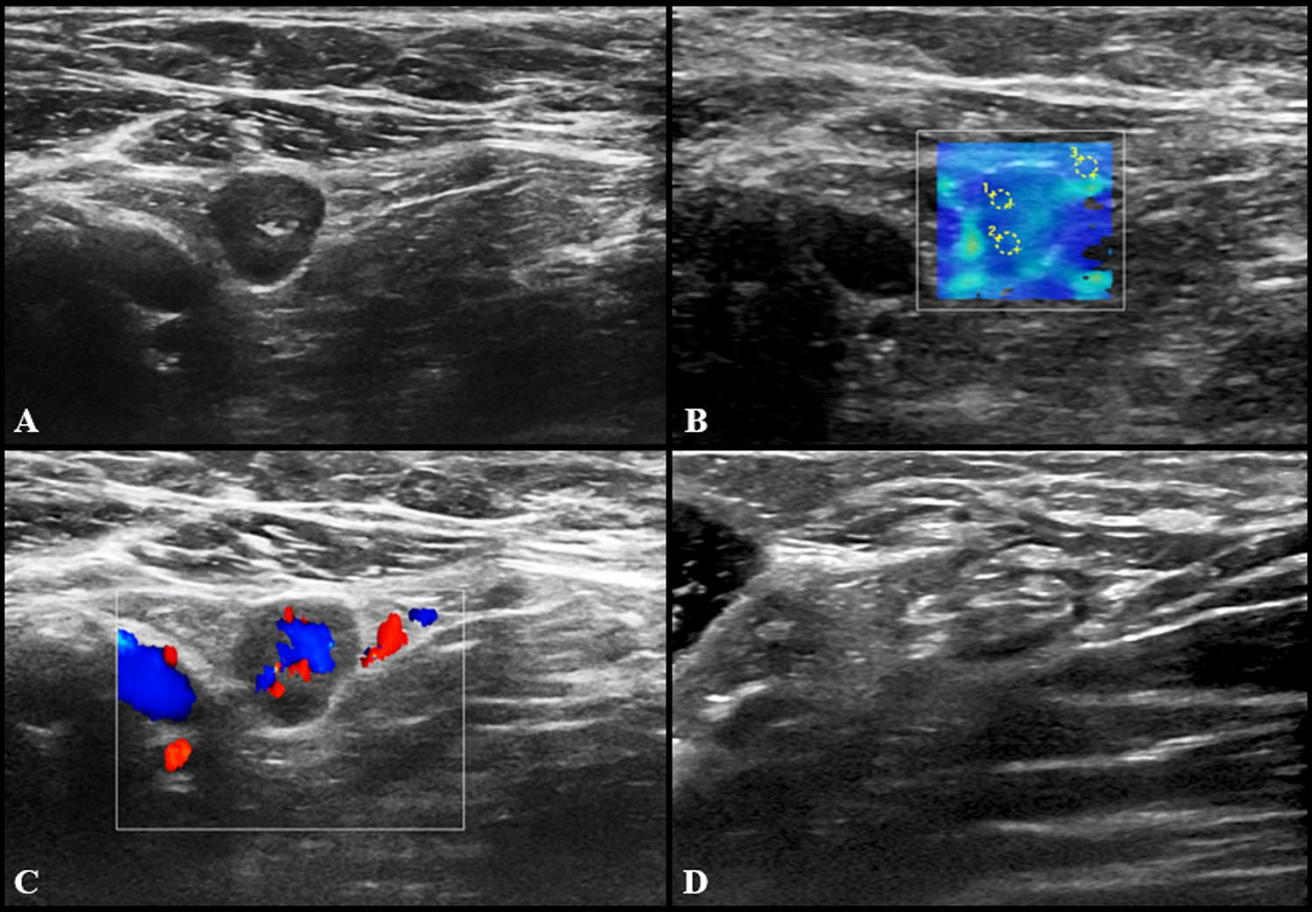
US scan performed in a 29-year-old woman 15 days after receiving the second dose of vaccine: axillary lymph node (8 × 9 mm) with fatty hilum but diffuse cortical thickening (**A**),and soft consistence at shear wave elastography (**B**: *k*1 = 7.50 kPa, *k*2 = 8.25 kPa and *k*3 = 7.4 kPa), and hilar vascularization (**C**) was detected. At the follow-up US scan performed after 10 weeks the lymph node reduction both in lymph nodes size and cortical thickness was observed (**D**)

**Fig. 6:**
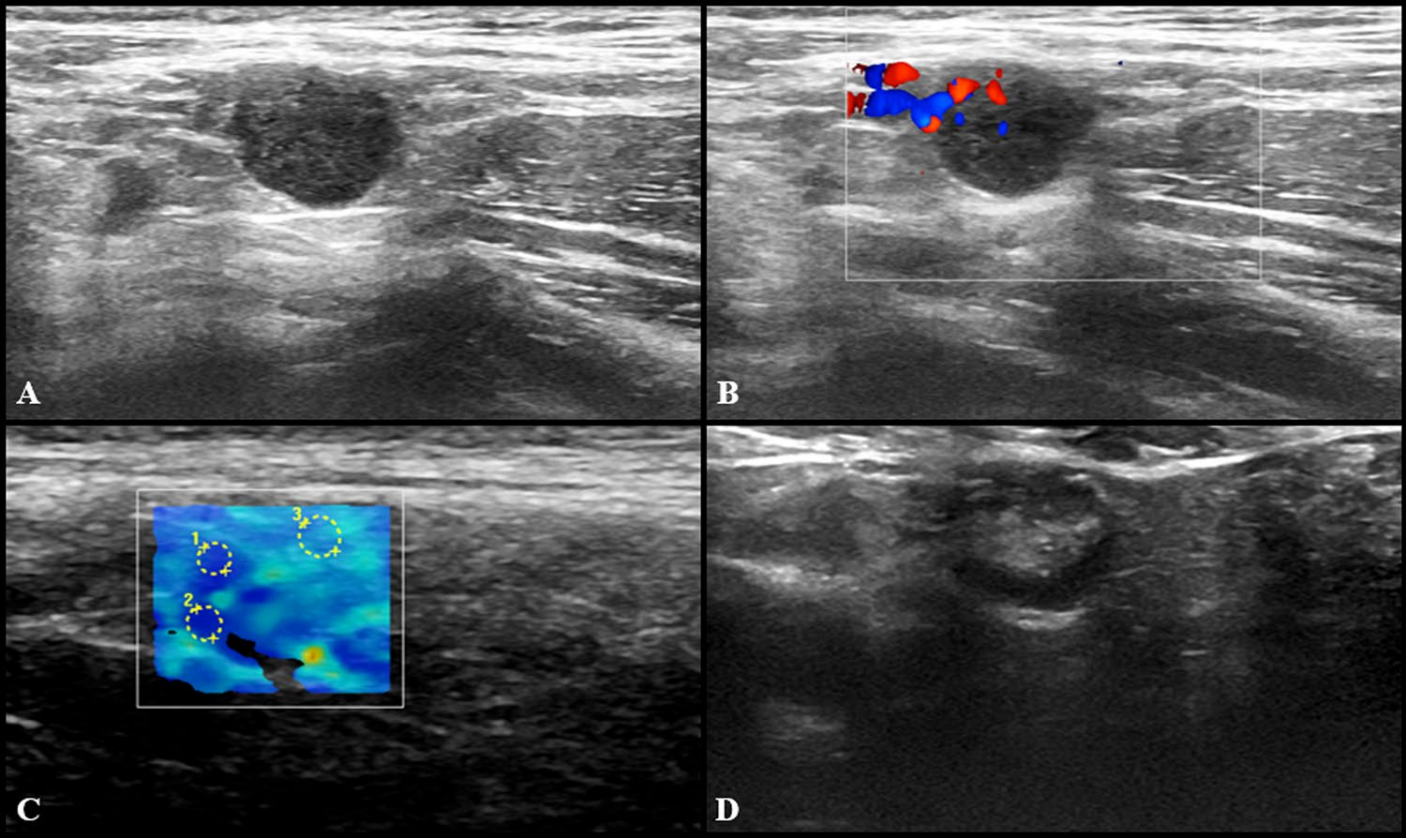
US examination performed in a 27-year-old woman 5 days after receiving the second dose of vaccine: a single round axillary lymph node with no detectable fatty hilum (**A**), cortical vascularization (**B**) and soft consistence at shear wave elastography (**C**: *k*1 = 5 kPa, *k*2 = 5 kPa and *k*3 = 11 kPa) was found. At the follow-up US scan performed after 4 weeks the lymph node appeared stable in size and shape but the fatty hilum was restored (**D**)

At US follow-up examinations, cortical thickness decreased in 8 subjects (11.6%), while in 54 of cases (78.2%) it was completely restored. The remaining 7 participants (10%) showed LNs of unchanged size, but no case has worsened. Follow-up examples of US lymphadenopathy are illustrated in Figs. 3, 4, 5 and 6.

## Discussion

In the last year, several studies on the occurrence and imaging features of post-COVID 19 vaccination axillary lymphadenopathy have been published, mostly during the last six months [2, 11, 16]. According to the Centers for Disease Control and Prevention, more than 11% of other vaccine cases lead to swollen LNs after one dose, rising to 16% after the second one [17]. Usually, they appear 2–4 days after the vaccine and decline after 2 weeks. Herein, we performed a prospective observational study to assess the rate and magnitude of Comirnaty-related axillary lymphadenopathy on US and the change in appearance and size of LNs over time. The characteristics of vaccine-associated lymphadenopathy may guide radiologists and physicians to rely on patient’s clinical context and updated resources to determine whether the findings can be considered suspected for malignancy [18]. This issue has to be considered relevant as, considering cyclic increase of COVID-19 cases due to the occurrence of viral variants (the Omicron one at the time of this writing), it cannot be excluded that additional doses of vaccine will be administered to the global population. Based on our previous experience, solitary and multiple axillary, subclavicular and latero-cervical LNs can be found abnormal at US examination performed after both first and second dose of Comirnaty, ipsilaterally to the site of injection, in young healthy subjects [6].

The currently administered Comirnaty vaccine provokes an intense immune response, due to both mRNA itself and lipid nanoparticles such as polyethylene glycol, all reported as highly immunogenic [11]. Due to this strong immune response, LN enlargement has been observed more than in previous cases of vaccine-associated lymphadenopathy [6, 8, 9, 19, 20], and particularly after the second dose. According to our findings, post-vaccine lymphadenopathy can occur in both symptomatic and asymptomatic subjects, and no correlations were found between their occurrence and any of the reported symptoms. It appears that the absence of armpit palpable lumps or pain does not exclude the presence of lymphadenopathy, even if a trend towards statistical significance was observed (*p* = 0.06). This finding suggests that a possible, intuitive correlation between this symptom and post-vaccine lymphadenopathy might have been missed due to the relatively small sample size. Main imaging findings consisted of cortical thickening, loss of fatty hilum and increased vascularization at color-Doppler. Taken together, such findings were similar to those of metastatic lymph-nodes, except for SWE of LN cortex, which showed median stiffness values (11 Kpa) lower than the currently reported cut-off values of malignant axillary LN [21].

Compared to other recently published studies, our work registered the highest incidence of vaccine-related lymphadenopathy (79.3%). Indeed, lymphadenopathy were classified as rare adverse events in 43.252 participants enrolled in a multinational, placebo-controlled trial study by Pollack et al. An incidence of 43% was reported by Skawran et al. (43%) on 90 positron emission tomography–computed tomography examinations. In a similar work including 169 patients, Eshet et al. revealed a 29% rate of positivity for LNs after the second dose of Comirnaty [2, 22]. Our results are in line with those reported by Faermann et al. in their study conducted in Israel, in which vaccination associated lymphadenopathy was attributed to 77.8% of cases, reaching values similar, albeit slightly inferior, to ours, thus confirming that the occurrence of post-vaccine lymphadenopathy has been initially underestimated [12].

While most of the previous experiences were mainly focused on describing incidence and imaging features of vaccine-related lymphadenopathy, data on follow-up imaging is currently still lacking. According to our findings, no significant US abnormalities were found at 4–12 weeks follow-up in the majority (78.2%) of cases, regardless of LN site, number, and appearance, whereas a remarkable decrease was detected in the remaining 8 subjects (11.6%), for a total regression rate of 89,8%. Such evidence confirms the hypothesis that enlarged LNs after Comirnaty can be considered not pathologic in the first instance, as already supported from literature [11, 23].

Our results reinforce the assumption that lymphadenopathy occurring ipsilaterally to the vaccination side after the second dose in subjects with no previous pathological conditions should be considered benign [24]. Therefore, short-term follow-up may be a suitable recommendation for those patients with specific clinical conditions (e.g. laboratory anomalies or oncological disease); additionally, in patients with previous history of breast cancer, it might be suggested to perform vaccine injection contralaterally to site of the previous breast malignancy. In this light, as also suggested by Faermann and colleagues, a deferral of any axillar imaging screening to at least 6 weeks after vaccination might also be proposed.

Some limitations of this study should be acknowledged. Firstly, the sample size was relatively small due and mainly included young health volunteers and such a cohort might not represent the overall population undergoing Comirnaty administration. Nevertheless, such a recruitment strategy allowed us to minimize the risk of incidentally reporting lymphadenopathy due to reasons other than COVID-19 vaccination. Secondly, the high variability encountered in the US scan time interval after second dose and for follow-up examination did not allow to perform additional formal analyses; while a more rigid time schedule could have been proposed, this would have affected the adherence to the study, thus possibly reducing the sample size.

In conclusion, a high incidence of axillary, supraclavicular and latero-cervical lymphadenopathy was found in a population of healthy young subjects, which showed a nearly complete regression within 4–12 weeks. Such findings should therefore be considered as benign, not deserving any additional follow-up.

## Supplementary Information

Below is the link to the electronic supplementary material.

**Supplementary Fig. 1** Patient selection flowchart. (TIFF 1449 KB)
