# Supplementary figures and images for: COVID-19 vaccine-induced lymphadenopathies: incidence, course and imaging features from an ultrasound prospective study
Source: J Ultrasound. 2022 May 4;25(4):965–71. doi: 10.1007/s40477-022-00674-3 (PMC9064721; doi:10.1007/s40477-022-00674-3)

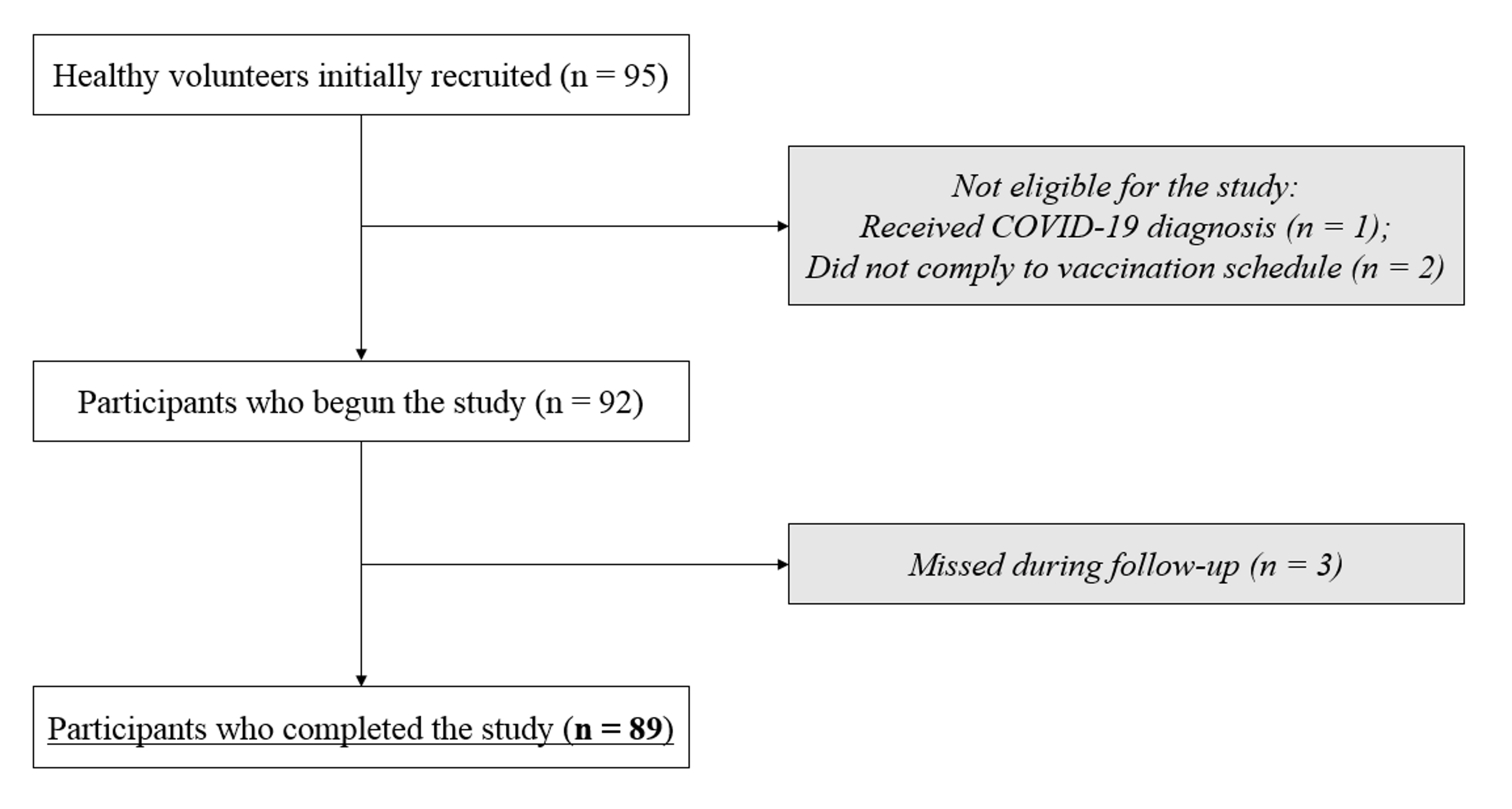

Supplement: Supplementary file 1 — Supplementary Fig. 1 Patient selection flowchart. (TIFF 1449 KB) [file 40477_2022_674_MOESM1_ESM.tiff]
